# LncRNA PITPNA-AS1 promotes gastric cancer by increasing SOX4 expression via inhibition of miR-92a-3p

**DOI:** 10.18632/aging.203403

**Published:** 2021-09-08

**Authors:** Licheng Liu, Anna Dai, Zao Zhang, Meiying Ning, Dong Han, Li Li, Zhuangzhuang Li

**Affiliations:** 1 Second Department of General Surgery, Cangzhou Central Hospital, Cangzhou, Hebei Province, China; 2 Meinian Health Clinic, Cangzhou, Hebei Province, China; 3 Department of Pharmacy, Cangzhou City Center Hospital, Cangzhou, Hebei Province, China

**Keywords:** gastric cancer, progression, lncRNA PITPNA-AS1, miR-92a-3p, SOX4

## Abstract

Background: Gastric cancer (GC) is a malignant tumor of digestive tract with high mortality. Elucidating the molecular mechanisms of GC and obtaining new molecular targets are particularly important for the prevention and treatment of GC. The discovery of long non-coding RNAs (lncRNAs) provides the possibility for further elucidating the molecular mechanisms of GC and discovering new molecular markers.

Aim: Here, we aimed to explore the function and the mechanism of lncRNA PITPNA-AS1 in GC.

Methods: High-throughput lncRNA microarray was used to compare the differences in expression profiles between tumor tissues and adjacent tissues, and to filtrate the differentially expressed lncRNAs in tumors. To analyze the relationship between lncRNA expression and clinicopathological parameters in GC. The apoptosis was detected by down-regulation of lncRNA. The effect of down-regulated lncRNA PITPNA-AS1 on the migration and invasion of GC cells was determined by wound healing and Transwell assays. The function of lncRNA PITPNA-AS1 on tumor growth was verified by tumor experiment in nude mice. Analysis of target interaction relationship was performed by luciferase assay.

Results: The results of high throughput chip analysis identified that PITPNA-AS1 was up-regulated in GC tissues. Our data revealed that knockdown of PITPNA-AS1 was able to inhibit tumor development of GC cells. Meanwhile, PITPNA-AS1 could regulate SOX4 expression via targeting miR-92a-3p.

Conclusion: Thus, we concluded that PITPNA-AS1 induced the development of GC cells by inhibiting miR-92a-3p and inducing SOX4. Our finding presents novel insights of GC, which may provide an underlying therapeutic target for GC treatment.

## INTRODUCTION

The incidence and mortality of gastric cancer (GC) rank first among gastrointestinal malignancy, which seriously threatens people's health and life. The pathogenesis of GC is closely related to multi-gene participation, multi-factor interaction and multi-step evolution [1, 2]. However, the molecular mechanism of GC still needs to be further studied.

LncRNAs are a kind of RNA, which has more than 200bp and cannot encode into protein. It plays many important roles in cells. At present, little is known about the role and related functions of lncRNAs in cells [3]. Previous studies have believed that lncRNAs are a transcriptional “impurity”. With the deepening of the study, it has been found that lncRNAs have many different biological functions in cells, and it shows low expression in a variety of malignant tumors, which participates in the tumors development. Intracellular miRNAs can target silencing gene expression, while lncRNA can inhibit gene silencing [4, 5]. LncRNA regulates gene expression by interacting with miRNA. LncRNA is the key endogenous RNA that regulates the interaction between miRNA and transcription factors [6, 7]. The imbalance of interaction between lncRNA, miRNA and protein can induce tumorigenesis [8]. Therefore, revealing the role of lncRNAs in tumors will provide the theoretical basis for elucidating its pathogenesis.

There are many kinds of abnormal expression of lncRNAs in GC, and many kinds of lncRNAs show low expression in GC, which is related to GC invasion, lymphatic and distant metastasis and TNM stage [5, 9]. After the high expression of lncRNA FENDRR in gastric cancer, the level of MMP2/MMP9 and FN1 is down-regulated, thus inhibiting cancer cell growth [10, 11]. There were differences in the expression of lncRNAs between serum and gastric juice, such as down-regulation of lncRNA HOTTIP and lnc-GNAQ-6:1 in serum of patients with GC [12, 13]. The expression of lncRNA H19 was abnormally increased in serum of patients with GC and down-regulated after operation. LncRNA-ANRIL contributes to the progression of gastric cancer by inducing NF-kB signaling [14]. LncRNA H19 directly induces the expression of ISM1 protein in GC, indirectly up-regulates miR-675 and then suppresses the expression of RUNX1 protein. LncRNA LINC00483 induces development of gastric cancer by modulating MAPK1 expression by targeting miR-490-3p [15]. LncRNA GAS5 inhibits cell proliferation via targeting E2F1 and P21 in gastric cancer and promoting apoptosis [16]. LncRNA MEG3 can regulate the expression of p53, induce apoptosis and inhibit cell proliferation in GC, which is associated with tumor size, depth of invasion and TNM stage [17].

In order to declare the role and underlying mechanism of lncRNA in GC, this study used high-throughput microarray to compare and determine the differences of lncRNA expression profiles between tumor and adjacent tissues, and uncover the role and mechanism of lncRNA PITPNA-AS1 in the progression of GC through multiple screening strategies, clinical sample analysis and functional loss assay.

## RESULTS

### PITPNA-AS1 is upregulated in GC tissues and cells

**Figure 1:**
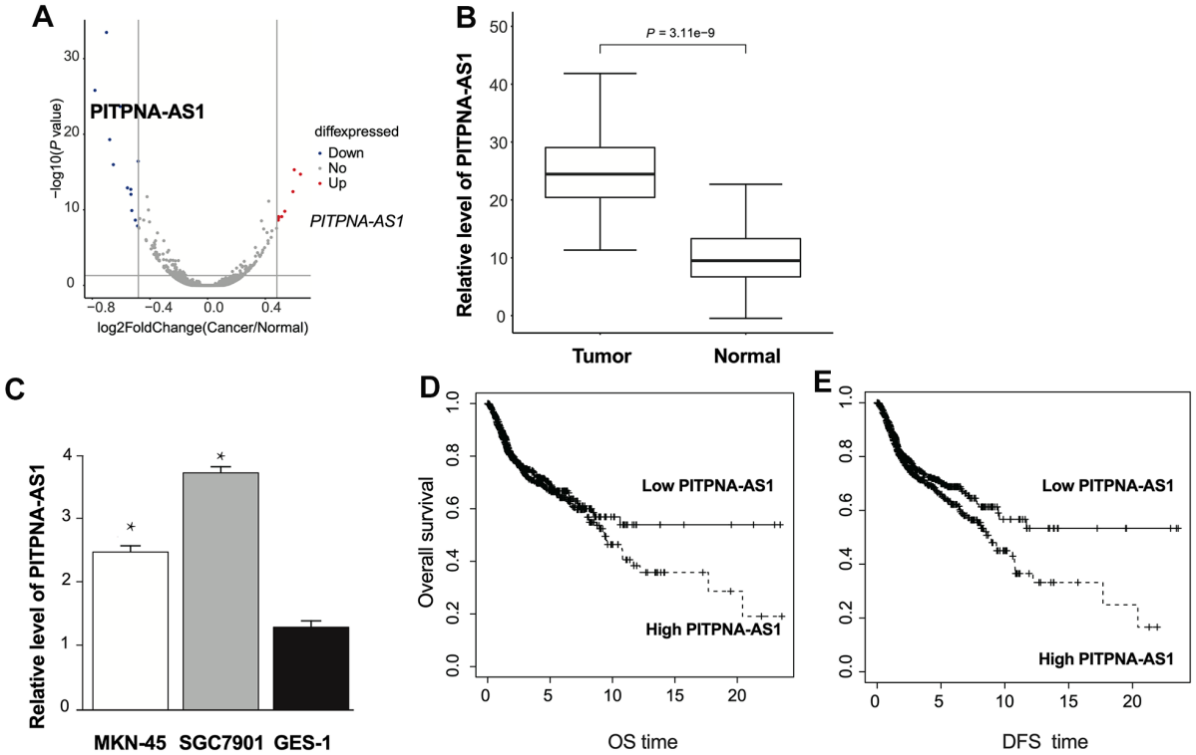
**The expression of PITPNA-AS1 is upregulated in GC patients.** (**A**) Volcano plot of the lncRNA profile in GC patients tumor and normal adjacent tissues. (**B**) The expression pattern of PITPNA-AS1 in GC patients tumor and normal adjacent tissues; (**C**) PITPNA-AS1 expression in MKN-45, SGC7901, and GES-1 cells; (**D**) dysregulation of PITPNA is related to overall survival in GC patients; (**E**) dysregulation of PITPNA is related to disease-free survival in GC patients. DFS: Disease-free survival; OS: Overall survival. Data are presented as mean ± SEM. Statistic significant differences were indicated: * P < 0.05, ** P < 0.01.

PITPNA-AS1 was found markedly increased in clinical GC tumor tissues, (Figure 1A). Further, qRT-PCR assay revealed that PITPNA-AS1 levels were increased in tumor tissues compared with normal adjacent tissues (Figure 1B). Further, PITPNA-AS1 expression was higher in GC cells (SGC7901 and MKN-45 cells) than GES-1 cells (Figure 1C). Furthermore, GC patients with a high level of PITPNA-AS1 had markedly shorter overall survival and disease-free survival than GC patients with a low level of PITPNA-AS1 (Figure 1D, 1E).

### Silencing of PITPNA-AS1 inhibits development and induces apoptosis of GC cells

**Figure 2:**
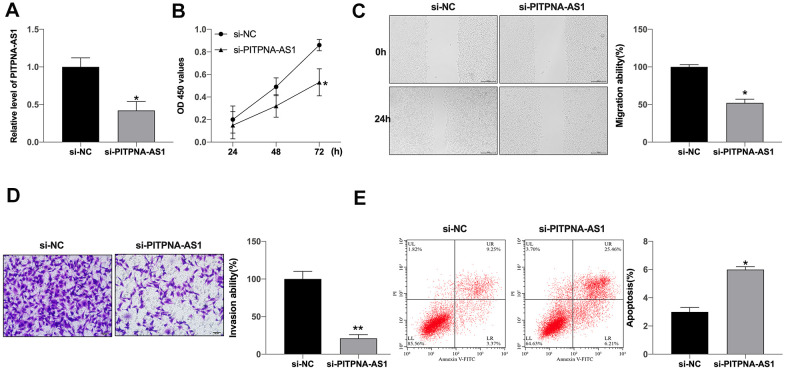
**PITPNA-AS1 regulates viability, migration, and invasion of GC cells.** (**A**) PITPNA-AS1 expression in SGC7901 cells with si-PITPNA-AS1 or si-NC; (**B**) PITPNA-AS1 siRNA inhibited cell proliferation in SGC7901 cells. PITPNA-AS1 siRNA inhibited SGC7901 cell migration (**C**) and invasion (**D**). (**E**) PITPNA-AS1 siRNA induced cell apoptosis in SGC7901 cells. Data are presented as mean ± SEM. Statistic significant differences were indicated: * P < 0.05, ** P < 0.01.

To explore the effect of PITPNA-AS1 on GC cells, we constructed the siRNA for silencing of PITPNA-AS1. The expression level of PITPNA-AS1 was decreased in SGC7901 cells after si-PITPNA-AS1 transfection (Figure 2A). The cell viability was inhibited by silencing of PITPNA-AS1 (Figure 2B). Consistently, wound healing assays demonstrated that si-PITPNA-AS1 remarkably inhibited the migration ability of SGC7901 cells (Figure 2C). Transwell assay performed that knockdown of si-PITPNA-AS1 prevented invasion ability of GC cells (Figure 2D). Moreover, apoptosis of SGC7901 cells was enhanced by si-PITPNA-AS1 transfection (Figure 2E), suggesting that knockdown of PITPNA-AS1 is able to inhibit development and induces apoptosis of gastric cancer cells.

### PITPNA-AS1 could target miR-92a-3p

**Figure 3:**
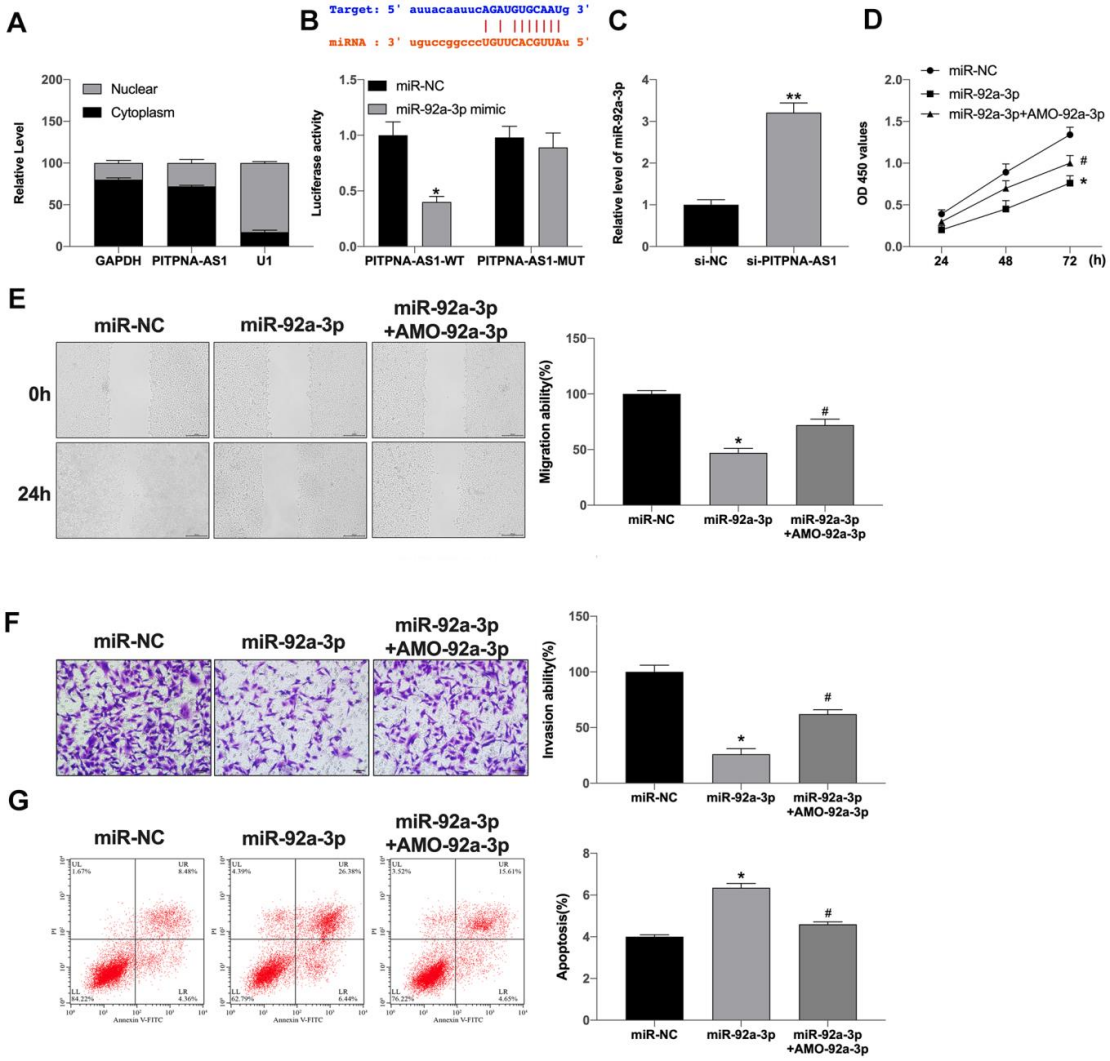
**PITPNA-AS1 acts as a sponge for miR-92a-3p in pancreatic carcinoma cells.** (**A**) Subcellular fractionation assay was performed to identify the location of PITPNA-AS1 in SGC7901 cells. (**B**) The binding sites between PITPNA-AS1 and miR-92a-3p; Luciferase activity of miR-92a-3p mimic with PITPNA-AS1-WT or PITPNA-AS1-MUT. (**C**) MiR-92a-3p expression is regulated by si-PITPNA-AS1 in SGC7901 cells. D-G: Cell proliferation (**D**), migration (**E**), invasion (**F**), and apoptosis (**G**) in SGC7901 cells treated with miR-92a-3p mimic and miR-92a-3p inhibitor (AMO- miR-92a-3p). Data are presented as mean ± SEM. Statistic significant differences were indicated: ns, no significance, * P < 0.05 vs. miR-NC, ** P < 0.01 vs. miR-NC; ^#^P <0.05 vs. miR-92a-3p.

Next, we were further explored the downstream of PITPNA-AS1 in regulating GC development. As Figure 3A shown that PITPNA-AS1 was mainly localized in cytoplasm. Bioinformatics website predicted that miR-92a-3p was a promising target of PITPNA-AS1, the predicted binding sequences were shown as Figure 2B. Luciferase assay reported declared the interaction between miR-92a-3p and a PITPNA-AS1 (Figure 3B). Silencing of PITPNA-AS1 promoted miR-92a-3p expression (Figure 3C). Further, forced expression of miR-92a-3p markedly inhibited cell viability, while was reversed by AMO-92a-3p (Figure 3D). In addition, wound healing assays revealed that miR-92a-3p remarkably decreased the wound proportion in SGC7901 cells. Transwell assays revealed that miR-92a-3p significantly attenuated the invasion of SGC7901 cells. However, AMO-miR-92a-3p blocked the function of miR-92a-3p on SGC7901 cells (Figure 3E, 3F). Moreover, miR-92a-3p induced the apoptosis level, which was abolished by the knockdown of miR-92a-3p (Figure 3G). Taken together, miR-92a-3p could regulate GC development.

### SOX4 is a downstream target of miR-92a-3p

**Figure 4:**
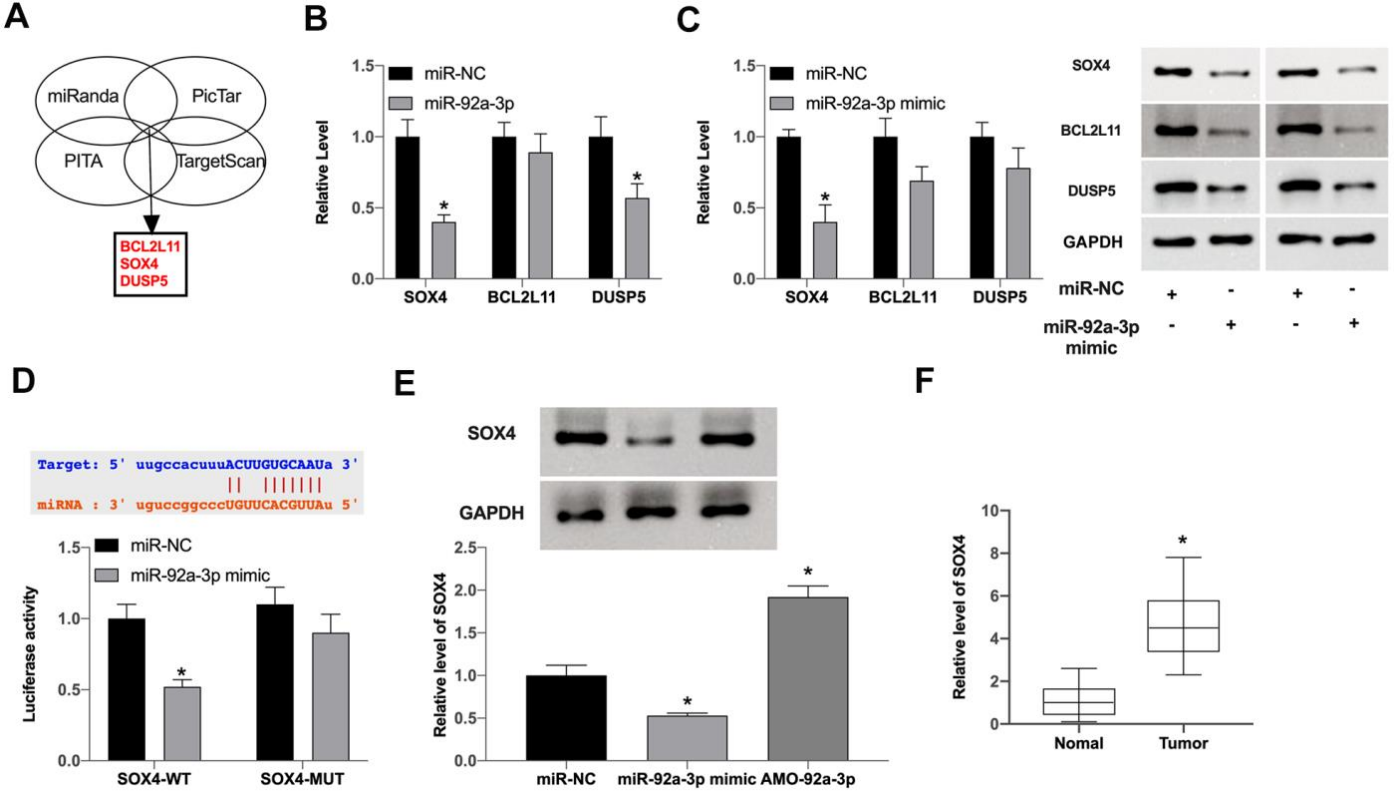
**MiR-92a-3p regulates SOX4 expression in pancreatic carcinoma.** (**A**) Three possible mRNAs regulated by miR-92a-3p were selected and are presented in a Venn diagram. (**B**, **C**) The expression levels of three target mRNAs were assessed after overexpression of miR-92a-3p. (**D**) The binding sites between SOX4 and miR-92a-3p; Luciferase activity of miR-92a-3p mimic with SOX4-WT or SOX4-MUT. (**E**) SOX4 expression regulation by miR-92a-3p mimic or inhibitor in SGC7901 cells. (**F**) SOX4 expression in pancreatic carcinoma tissues. Data are presented as mean ± SEM. Statistic significant differences were indicated: * P < 0.05.

Next, we would explore the downstream target of PITPNA-AS1/miR-92-3p. Bioinformatics website predicted that BCL2L11, SOX4, and DUSP5 were the underlying target of miR-92a-3p (Figure 4A). Then we determined the level of BCL2L11, SOX4, and DUSP5 in GC cells after miR-92a-3p, we found that sox4, not BCL2L11 or DUSP5 was significantly downregulated in GC cells (Figure 4B, 4C). Luciferase assay verified that SOX4 could bind with miR-92a-3p (Figure 4D). Then we detected the protein level of SOX4 in GC cells after the gain and loss function of miR-92a-3p. Forced expression of miR-92a-3p prevented SOX4 level, and AMO-92a-3p induced the expression of SOX4 (Figure 4E). Further, we detected the expression of SOX4 in clinical GC tumor tissue and normal tissues, the increased level of SOX4 was found in tumor tissues (Figure 4F). In summary, SOX4 was a downstream target of miR-92a-3p.

### PITPNA-AS1 regulates GC cells development via miR-92a-3p/SOX4 signal

**Figure 5:**
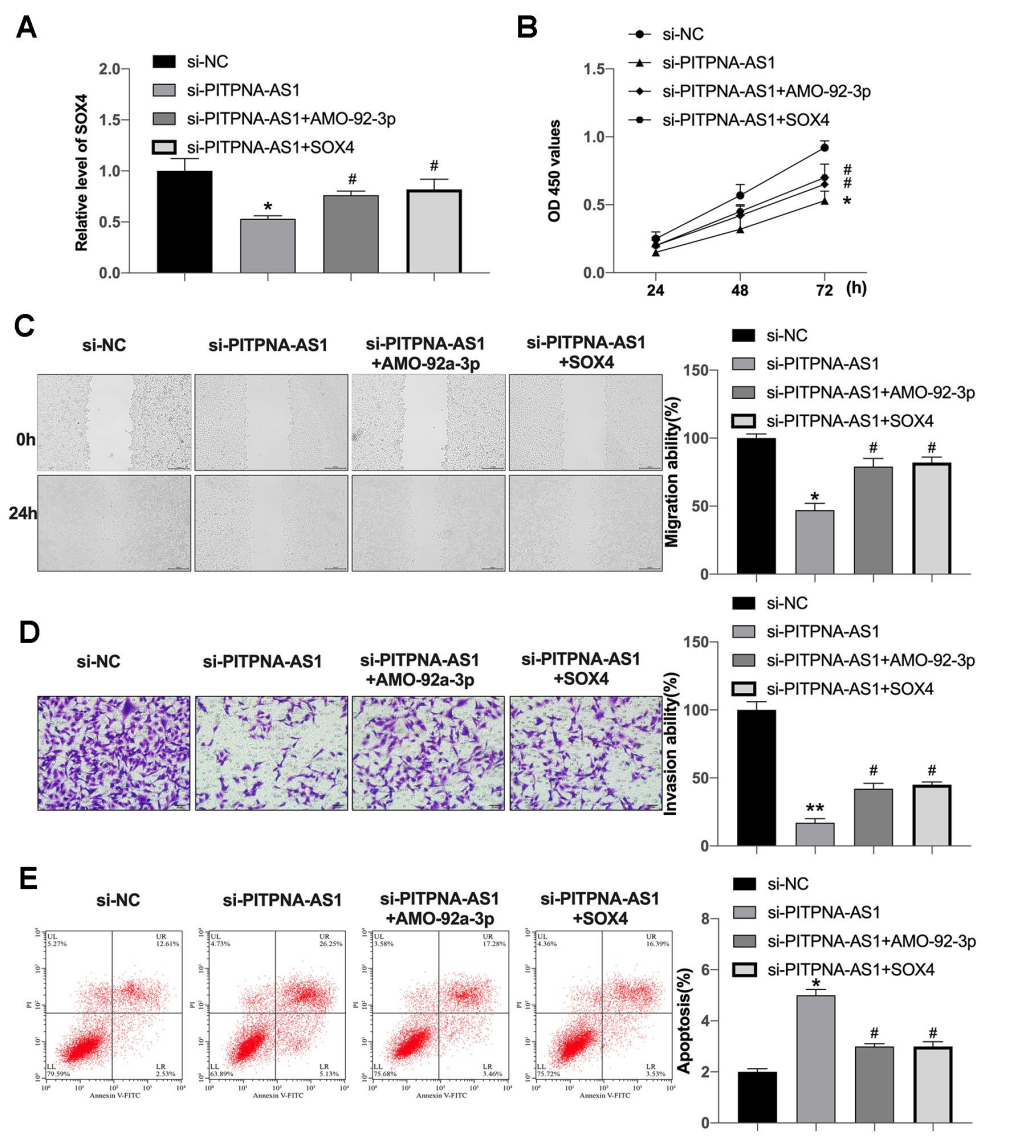
**PITPNA-AS1 interacts with miR-92a-3p to regulate SOX4 expression.** (**A**) SOX4 expression in SGC7901 cells; (**B**–**E**) cell proliferation (**B**), migration (**C**), invasion (**D**), and apoptosis (**E**) in SGC7901 cells containing si-PITPNA-AS1, miR-92a-3p mimic, and SOX4 vector. Data are presented as mean ± SEM. Statistic significant differences were indicated: ** P < 0.01 vs. si-NC; ^#^P <0.05 vs.si-PITPNA.

Further, we constructed SOX4 plasmid and miR-195-5p mimic, which were co-transfected into GC cells. The expression of SOX4 was detected by qRT-PCR (Figure 5A). Silencing of PITPNA-AS1 inhibited cell viability which was prevented by AMO-miR-92a-3p and SOX4 plasmid (Figure 5B). Further, silencing of PITPNA-AS1 inhibited migration of GC cells, in which the treatment of AMO-miR-92a-3p or SOX4 overexpression could reverse the effect in the cells (Figure 5C). Consistently, the invasion ability of GC cells was inhibited by PITPNA-AS1 depletion, while the AMO-miR-92a-3p or SOX4 overexpression was able to restore the phenotype in the cells (Figure 5D). PITPNA-AS1 knockdown also induced apoptosis level, AMO-miR-92a-3p and SOX4 blocked the effect of si-PITPNA-AS1 in GC cells (Figure 5E).

### PITPNA-AS1 involves in tumor growth *in vivo*

**Figure 6:**
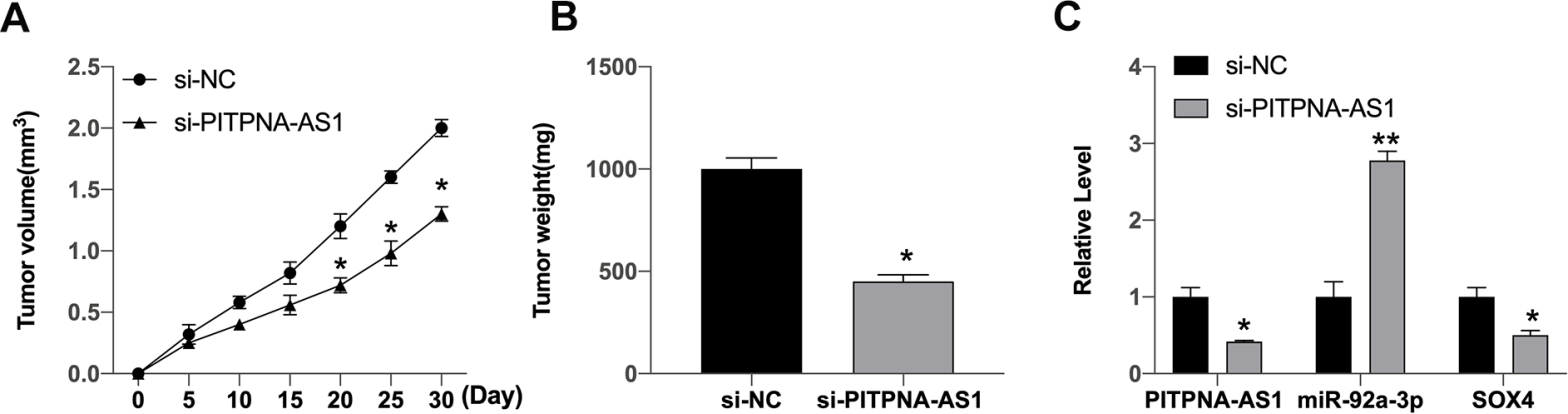
**PITPNA-AS1 regulates the tumor growth *in vivo*.** (**A**, **B**) The impact of si-PITPNA-AS1 on tumor growth of gastric cells *in vivo* was analyzed by nude mice tumorigenicity assay (n = 5). (**C**) The expression of PITPNA-AS1, miR-92a-3p, SOX4 was determined. Data are presented as mean ± SEM. Statistic significant differences were indicated: * P < 0.05.

We constructed stable PITPNA-AS1 low expression SGC7901 cells and injected them into nude mice to observe the tumor growth. After 30 days, silencing of PITPNA-AS1 prevented tumor volume and weight (Figure 6A, 6B). Then we isolated tumor tissues to assess the level of PITPNA-AS1, SOX4 and miR-92a-3p (Figure 6C).

## DISCUSSION

At present, the treatment of GC is still surgery supplemented by radiotherapy and chemotherapy. Although surgery can remove the primary focus, it is difficult to achieve a radical cure. Postoperative recurrence and distant metastasis of GC patients are the main reasons for their low long-term survival rate. Therefore, studying the molecular mechanism of invasion and metastasis of GC and finding new prognostic markers and therapeutic targets would be helpful to the treatment and prognosis monitoring of GC. In the present, we discovered the critical role of PITPNA-AS1 in the modulation of GC development by targeting miR-92a-3p/SOX4 axis.

The previous studies have shown that lncRNA is closely associated with GC development. Many lncRNAs are differentially expressed in GC tissues or plasma of patients with GC, and their expression levels are also related to the clinicopathological features of GC, which may be used as molecular markers for early diagnosis, progression and prognosis monitoring of GC [18, 19]. Accordingly, it can explore new molecular markers related to GC by studying the differentially expressed lncRNA in GC. It has been reported that PITPNA-AS1 is located in chromosome 17p13.3 [20]. In previous research, PITPNA-AS1 could affect hepatocellular carcinoma development [21]. Furthermore, PITPNA-AS1 could regulate WNT5A via sponging miR-876-5p. PITPNA-AS1 also regulates cervical cancer progression via targeting miR-876-5p and PITPNA-AS serves as a target for cervical cancer [22]. PITPNA-AS1 also regulates lung squamous cell carcinoma cell development. PITPNA-AS1 recruited TAF15 to maintain HMGB3 in lung squamous cell carcinoma cells [23]. These previous studies indicate that PITPNA-AS1 may serve as a oncogenetic factor in cancer progression. Here, we revealed that PITPNA-AS1 was markedly upregulated in GC tissues, high level PITPNA-AS1 was negatively correlated with overall survival and disease-free survival. Silencing of PITPNA-AS1 prevented GC cells cell viability, migration ability, and invasion ability. Luciferase assay confirmed that miR-92a-3p could interact with PITPNA-AS1. These data suggest that PITPNA-AS1 contributes to GC development by targeting miR-92a-3p, providing crucial evidence of the important function of PITPNA-AS1 in cancer progression. Meanwhile, we innovatively identified the correlation of PITPNA-AS1 with miR-92a-3p in GC development. The function of PITPNA-AS1/miR-92a-3p axis in other cancers should be explored by more investigations. The clinical significance of PITPNA-AS1 and miR-92a-3p need to be confirmed in future studies.

SOX4 belongs to the family of SOX transcription factors, which plays a crucial role in the formation of heart, the development and differentiation of T B lymphocytes, the development of pancreas and the development of bone during embryonic development [24, 25]. SOX4 is a transcription factor with multiple biological functions. SOX4 gene encodes a protein of 474 aa and is also a single exon gene. In recent years, more and more studies have shown that the expression of SOX4 is increased in many human tumors and is closely related to the development of tumors [26, 27]. In different types of tumors or different stages of tumors, SOX4 plays a dual role by participating in the process of tumor cell proliferation, apoptosis or differentiation: tumor promotion and tumor inhibition. At present, some scholars have found the abnormal expression of SOX4 in many kinds of human malignant tumors, such as breast cancer, bladder cancer, ovarian cancer, colorectal cancer, prostate cancer, hematological malignant tumor and so on [24, 25, 27–29]. The abnormal expression of SOX4 is significantly related to tumor characteristics and prognosis of patients [24, 25, 27–29]. Meanwhile, it has been reported that lncRNA ZFAS1 contributes to the metastasis of colorectal cancer by regulating miR-34b/SOX4 axis [30]. Here, we found that PITPNA-AS1 could regulate SOX4 expression via sponging miR-92a-3p. This new lncRNA of PITPNA-AS1 should be assessed as prognostic factor to influence therapeutic strategies and more clinical investigations should be performed. These findings indicate that PITPNA-AS1 induces SOX4 expression by sponging miR-92a-3p to promote GC progression. Our finding provides new insight into the mechanism by which PITPNA-AS1 regulates GC through miR-92a-3p/ SOX4 signaling. SOX4 may be just one of the targets of PITPNA-AS1/miR-92a-3p axis in GC development and other downstream factors should be explored in future investigations.

## CONCLUSIONS

In summary, we concluded that PITPNA-AS1 could target miR-92a-3p/SOX4 signal pathway, which may serve as the potential therapeutic candidate for GC treatment.

## MATERIALS AND METHODS

### Clinic sample

42 cases of gastric cancer tissues and normal mucosal tissues more than 5 cm apart from the tumor harvested from removed operative specimens were collected. All the cases were confirmed as gastric adenocarcinoma by pathological diagnosis, except for patients with other malignant tumors and patients who received radiotherapy, chemotherapy and other treatment for gastric cancer before operation. The specimens used in the experiment were discussed and approved by the ethics committee of Cangzhou City Center Hospital, and the informed consent of the patients was obtained.

### Cell culture and treatment

SGC7901 cell line was cultured at the incubator of 5% CO_2_ and 37° C with the 1640 medium (Hyclone, USA) with FBS (10%, Hyclone, USA), streptomycin (0.1 mg/mL, Hyclone, USA) and penicillin (100 units/mL, Hyclone, USA).

### Transwell assays

Transwell assays analyzed invasion of GC cells by using a Transwell plate (Corning, USA) according to the manufacturer's instruction. Shortly, the upper chambers were plated with around 1 × 10^5^ cells. Then solidified through 4% paraformaldehyde and dyed with crystal violet. The invaded and migrated cells were recorded and calculated.

### Wound healing assay

Cells were plated in the 24-well plate at 3 × 10^5^/well and cultured overnight to reach a full confluent as a monolayer. A 20μl pipette tip was applied to slowly cut a straight line across the well. Then the well was washed by PBS 3 times and changed with the serum-free medium and continued to culture. The wound healing percentage was calculated.

### CCK-8

The cells were seeded into 96-well plate. After transfection, 10 μ L / well of CCK-8 was added into cells for 2 hours. The absorbance of A450 nm was detected by full-band enzyme labeling MULTISKAN GO (Thermo).

### Western blot

The cell and tissue samples were washed with pre-cooled phosphate-buffered saline (PBS) and then lysed with cell lysis solution (RIPA; Beyotime). Protein concentration was detected using the bicinchoninic acid (BCA; Thermo Fisher Scientific Inc., USA). The proteins were transferred onto a polyvinylidene difluoride (PVDF) membrane (Millipore, Billerica), blocked in PBS containing 5% skimmed milk, and incubated for 2 h. The proteins were incubated with the primary antibody of target proteins and incubated at 4° C overnight. After being washed (3×10 min) with PBST, the secondary antibody was added and incubated at room temperature for 1 h. Results were analyzed by the Image J software.

### Transwell assay

A Transwell chamber (8.0 μm aperture) was used to evaluate the invasive ability of cells. The transfected cells were re-suspended with a serum-free medium after overnight starvation. 100 μL single-cell suspension containing 2x10^4^ cells was seeded into the upper cavity pre-covered with Matrigel matrix glue, and the lower cavity was added with human fetal bovine serum 600 μL for 48 hours. The cells were fixed and stained with crystal violet, and the invading cells were counted randomly in each sample.

### Wound healing assay

The transfected cells were seeded into a 6-well plate (1x10^5^/ well). The cells were routinely cultured in the incubator until the cells converged. The monolayer of cells was scratched with a 200 μL pipette, and the scratched cells were washed and removed by PBS. After 24 hours of culture, regions were randomly selected by an inverted microscope to obtain images and measure the width of scratches.

### Flow cytometry

The transfected cells were collected. Human Annexin V-FITC5 μL was added to the cell suspension, 10 ug/mL propidium iodide (PI) 10 μL, 4 C light avoidance reaction 15 min, and 4° C mixed buffer 200 μL were added. The cell apoptosis rate were detected by the Guava Nexin test according to the manufacturer’s instructions (Millipore/Guava Technologies, USA).

### Analysis of cell apoptosis

Cell apoptosis was measured by applying the Annexin-V-FITC apoptosis kit (BD, USA) based on flow cytometry analysis using FACSCalibur flow cytometer, followed by the quantification analysis of FlowJo software.

### Xenograft tumor model

stabled PITPNA-AS1 low expression SGC7901 cells or normal cells were transfected in the logarithmic phase were collected and centrifuged, and washed into cell suspension (2x10^7^/mL). 3-4 week male BALB/e (nu/nu) nude mice were randomly divided into different groups and subcutaneously injected with 0.2 mL of cell suspension on the back of the right hindlimb. The short diameter (A) and long diameter (B) of the tumor were measured with Vernier caliper every 5 days after inoculation. The tumor volume was calculated as BA^2^/2. The mice were observed continuously for 30 days. The animal study was reviewed and approved by Cangzhou City Center Hospital.

### Statistical analysis

Prism 8.0 software was used for statistical analysis; the data were expressed as mean ±SEM. T-test was used for the comparison between the two groups, and one-way ANOVA was used for the comparison among the groups. *P* < 0.05 was statistically significant, and the experiments were repeated more than 3 times.
